# Rising Deaths due to Malnutrition and Growing Disparities in the U.S.: A 24‐Year Trend Analysis From 1999 and 2023

**DOI:** 10.1002/fsn3.71040

**Published:** 2025-10-22

**Authors:** Shree Rath, Ghazal Ishaque, Saif Ur Rahman, Ahmad Sanan, Bushra Syed, Mubashira Ali, Pinkey Kumari, Amar Lal

**Affiliations:** ^1^ All India Institute of Medical Sciences Bhubaneswar Bhubaneswar India; ^2^ Shaheed Mohtarma Benazir Bhutto Medical College Karachi Pakistan; ^3^ Bacha Khan Medical College Mardan Pakistan; ^4^ Khyber Medical College Peshawar Pakistan; ^5^ Jinnah Medical and Dental College Karachi Pakistan; ^6^ Hamdard College of Medicine and Dentistry Karachi Pakistan; ^7^ Penn State Health Milton S Hershey Medical Center Pennsylvania State University Hershey Pennsylvania USA

**Keywords:** epidemiology, geriatric, malnutrition, public health, racial disparities

## Abstract

Malnutrition is an acutely growing public health concern in the U.S., with over 20% predicted to be malnourished by the year 2030. This study aims to analyze disparities in malnutrition‐related mortalities in the United States from 1999 to 2023. A descriptive study using data from the CDC WONDER database was conducted to examine trends and disparities in malnutrition‐related deaths in adults aged 55–85 years and older. The study classified deaths by age, gender, race, place of death, and urban–rural classification. Age‐adjusted mortality rates (AAMRs) and crude mortality rates per 100,000 population were estimated, and Joinpoint regression analysis was applied to identify annual percent changes (APCs) and average annual percent changes (AAPCs). From 1999 to 2023, 158,117 deaths due to malnutrition were recorded among individuals aged 55 and above. Initial trends showed a decline with an APC of −8.38% from 1999 to 2006, followed by a significant rise from 2013 to 2021 with an APC of 22.51%. The oldest age group showed the highest annual rise of 5.74%. Gender‐specific trends revealed a steep rise in malnutrition‐related deaths for both males and females from 2013 to 2023. Racial disparities showed significant increases in mortality rates among White individuals, with an AAPC of 5.91%. The study reveals a significant resurgence of malnutrition‐related mortality among older adults in the U.S. after years of decline, highlighting the need for targeted public health interventions. Geographical, gender‐specific, and racial disparities suggest systemic healthcare issues that require gender‐sensitive and culturally appropriate nutritional screening and management strategies.

## Graphical abstract

This study analyzes malnutrition‐related deaths in the U.S. between 1999 and 2023, revealing rising mortality rates among older adults after 2013. Significant disparities in malnutrition‐related deaths were found across age groups, genders, races, and geographic regions, underscoring the need for targeted public health interventions.

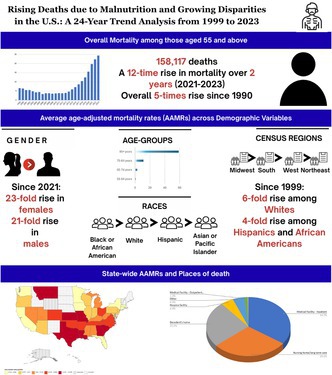

## 1 Introduction

Malnutrition is defined as a decrease in muscle and cell mass caused by an insufficient amount of nutrient intake or absorption, which leads to impaired mental and physical function, reduced quality of life, and poor health outcomes (Serón‐Arbeloa et al. 2022). Malnutrition is characterized by weight loss, diminished physical strength, decreased appetite, and poor dietary intake (Salari et al. 2025). Despite being widespread, malnutrition often goes undiagnosed due to the absence of clear diagnostic criteria, making it difficult to detect in both hospital and community settings (Chamanoor et al. 2024). It is increasingly recognized as a global health concern due to its serious effects, including the worsening of underlying conditions and increased mortality risk (Chamanoor et al. 2024). Age is one of the key risk factors for disease development, and the prevalence of malnutrition is increasing among older adults. Involuntary weight loss or a low body mass index are common signs associated with aging. Still, hidden deficiencies such as micronutrient deficiencies are more difficult to diagnose and are often overlooked, especially among older individuals living in the community. In underdeveloped countries, disease is the most frequently reported cause of malnutrition, as both acute and chronic illnesses can lead to or worsen nutritional deficiencies (Norman et al. 2021).

With the aging population on the rise, it's estimated that over 25% of older adults are experiencing malnutrition (Mostafa et al. 2023). As global life expectancy increases, the risk of malnutrition among older adults is also expected to rise (Prell and Perner 2018). In the United States, the number of older individuals is expected to reach 72 million by 2030, which will comprise around 20% of the overall population (White et al. 2018).

This study aims to analyze disparities in malnutrition‐related mortalities in the United States from 1999 to 2023, using data from the Centers for Disease Control and Prevention's Wide‐Ranging Online Data for Epidemiologic Research (CDC WONDER) database. We will examine trends in mortality across a range of settings, including nursing homes, medical facilities, home/hospice care, and other locations. The study focuses on demographic variations in the site of death based on variables such as age, gender, race, and census region. We will also examine potential causes of the observed gaps and suggest how these findings can be used to better understand and manage malnutrition‐related mortality in the US.

## 2 Methods

### 2.1 Study Setting and Population

In this descriptive study, death certificate data were collected from the CDC WONDER (Centers for Disease Control and Prevention Wide‐Ranging Online Data for Epidemiologic Research) database (CDC WONDER 2025) and analyzed between 1999 and 2023 to examine trends and disparities in malnutrition‐related death in adults aged 25–85 years and older. Deaths were defined by the International Statistical Classification of Diseases and Related Health Problems, 10th Revision (ICD‐10) codes E40–E46, which correspond to protein‐energy malnutrition, such as kwashiorkor, marasmus, and other severe nutritional deficiencies. These codes have been applied before to estimate malnutrition‐related death in national epidemiologic studies (Mostafa et al. 2023) The dataset comprises cause‐of‐death data from death certificates reported in all 50 U.S. states and the District of Columbia. The Multiple Cause‐of‐Death Public Use file was employed to select cases in which malnutrition was noted as the underlying or a contributing cause of death. Adults were classified as individuals aged 55 years and older at the time of death and were divided into the following age groups: 55–64, 65–74, and 75–85 years and older, as in previous analyses of adult mortality. This research was exempt from institutional review board approval since it employed publicly available, deidentified data and conformed to the STROBE (Strengthening the Reporting of Observational Studies in Epidemiology) guidelines (von Elm et al. 2007).

### 2.2 Data Extraction

Data were extracted for population size, year of death, place of death, demographic characteristics, urban–rural classification, geographic region, and state. The demographic information included age, sex, and race/ethnicity. The place of death included medical facilities (outpatient, emergency department, inpatient, death on arrival, or unknown), home, hospice, and long‐term care or nursing home institutions. Race/ethnicity was defined as non‐Hispanic (NH) White, NH Black or African American, Hispanic or Latino, NH American Indian or Alaskan Native, and NH Asian or Pacific Islander, based on death certificate information (Centers for Disease Control and Prevention, National Center for Health Statistics, n.d.) The National Center for Health Statistics Urban–Rural Classification Scheme was used to classify counties as urban (large metropolitan area [population ≥ 1 million], medium/small metropolitan area [population 50,000–999,999]) or rural (population < 50,000), using the 2013 U.S. Census classification (Ingram and Franco 2014) U.S. geographic regions were defined as Northeast, Midwest, South, and West, based on the standards established by the U.S. Census Bureau.

### 2.3 Statistical Analysis

To evaluate the national trends in malnutrition‐related mortality, age‐adjusted mortality rates (AAMRs) and crude mortality rates per 10,000 population were estimated between 1999 and 2023 with stratification by year, race/ethnicity, sex, state, and urban–rural classification, based on 95% confidence intervals (CIs). Crude mortality rates were estimated by tabulating malnutrition deaths and dividing them by the respective U.S. population for the year. AAMRs were estimated through the direct method and standardized to the 2000 U.S. standard population. Joinpoint regression analysis was applied using the Joinpoint Regression Program (version 4.9.0.0, National Cancer Institute) for the analysis of trends in mortality, to estimate annual percent change (APC) (National Cancer Institute, n.d.) and to calculate statistically significant changes in trends. Joinpoint regression uses log‐linear regression models and identifies inflection points where trends significantly alter. An APC was considered significant if the slope differed from zero, as derived based on 2‐tailed *t*‐tests. The significance level was *p* < 0.05.

## 3 Results

### 3.1 Malnutrition‐Related AAMR—Overall

**FIGURE 1:**
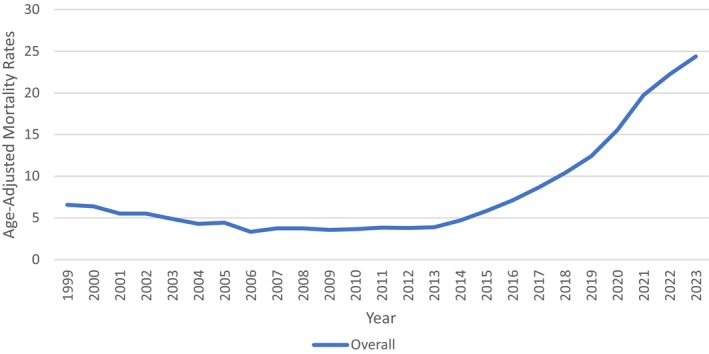
Overall malnutrition‐related age‐adjusted mortality rate per 100,000 in the United States, 1999–2023.

From 1999 to 2023, there were a total of 158,117 deaths due to malnutrition among individuals aged 55 years and above. Trend analysis initially showed a declining trend with an APC of −8.380 (95% CI: 11.2956–6.7057) from 1999 to 2006, and another significant trend was observed from 2013 to 2021 with an APC of 22.51 (95% CI: 21.6988–24.2644). Further rates inclined from 2021 to 2023 with an APC of 12.70 (95% CI: 9.6592–16.6811). These trends yield an overall AAPC of 5.4991 (95% CI: 5.1996–5.8895). The results were further categorized by age, gender, race, census, urbanization, place of death, and state. (Figure 1 and Figure S1, Tables S1 and S2).

### 3.2 Malnutrition‐Related AAMR—Stratified by 10‐Year Age Groups

In the 55–64 age group, there were 3 joinpoints identified, and the rate declined initially from 1999 to 2005, but then increased from 2005 to 2023 with a prominent spike from 2013 to 2023 with an APC of 13.3426 (95% CI: 12.2956–15.11), yielding an overall AAPC of 5.6374 (95% CI: 5.0777–6.3367). Among those 65–74, the rate fell by −5.5114 (95% CI: −16.3865 to 1.0542) across the period (1999–2006) which then increased continuously from 2006 to 2023 with a prominent spike from 2013 to 2023 with an APC value of 17.1519 (95% CI: 15.34–21.78), the overall trend yielding an AAPC of 5.723 (95% CI: 5.134–6.7828) during the entire period. For the age group 75–84 years, 4 different trends were observed where the trend initially decreased from 1999 to 2006, which then increased continuously from 2006 to 2023 with a prominent spike from 2014 to 2021, with an APC of 21.6042 (95% CI: 19.9561–27.1662) yielding an overall AAPC of 4.989 (95% CI: 4.580–5.534). For the age group 85+ years, there were 4 different trends with a similar initial declining trend that was followed by a surge in the trend of malnutrition with a significant spike from 2013 to 2021 with an APC of 25.6857 (95% CI: 24.5363–29.0922) that yielded an overall AAPC of 5.7376 (95% CI: 5.3048–6.2921) from 1999 to 2023 (Figure S2, Table S3).

### 3.3 Malnutrition‐Related AAMR—Stratified by Gender

**FIGURE 2:**
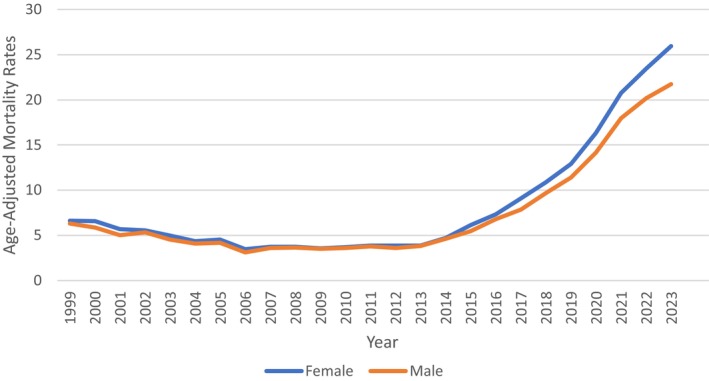
Malnutrition‐related age‐adjusted mortality rate per 100,000, stratified by gender in the United States, 1999–2023.

Among females, 4 different trends were observed across the entire period where the rate dropped from 1999 to 2007 with an APC of −7.6883 (95% CI: −9.8206 to 6.3113), which was followed by an increasing trend till 2023 along with a prominent spike from 2013 to 2021 with an APC of 22.9603 (95% CI: 22.1493–24.8794) that resulted in the final AAPC of 5.7489 (95% CI: 5.4652–6.1049) from 1999 to 2023 for females. Males' trends of malnutrition declined similarly from 1999 to 2006, followed by an ever‐increasing trend till 2023 with a significant spike from 2013 to 2021 with an APC value of 21.4335 (95% CI: 20.5312–23.8744) that yields an overall AAPC of 5.3107 (95% CI: 4.9593–5.7544) from 1999 to 2023 for males (Figure 2, Figure S3, Table S2).

### 3.4 Malnutrition Related AAMR—Stratified by Race

**FIGURE 3:**
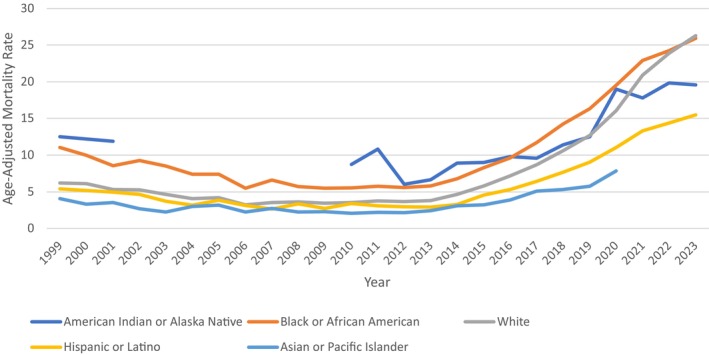
Malnutrition‐related age‐adjusted mortality rate per 100,000, stratified by race in the United States, 1999–2023.

In Black or African American individuals, 4 different trends were observed. Initially, there was a declining trend from 1999 to 2009 with an APC of −6.6621 (95% CI: −9.4771 to 5.5638) that was followed by an insignificantly increasing trend from 2009 to 2023, which yielded an overall AAPC of 3.7203 (95% CI: 3.3675–4.0955) from 1999 to 2023. Among White individuals, the rate fell from 1999 to 2006 at −8.3604 (95% CI: −10.957 to 6.7533), which was followed by a continuous increasing trend, spiking between 2013 and 2021 (APC: 23.6745′ 95% CI: 22.8086, 25.5863) and from 2021 up to 2023 that yielded an overall AAPC of 6.0557 (95% CI: 5.7538–6.4368) from 1999 to 2023. Among Hispanic or Latino individuals, there were similar 4 identified joinpoints, which declined in value initially, followed by an ever‐increasing trend till 2023 with a significant spike from 2013 to 2021 with an APC value of 20.4535 (95% CI: 19.2585–22.8408), making the AAPC for the entire interval 4.3263 (95% CI: 3.8544–4.8773) from 1999 to 2023 (Figure 3, Figure S4, Table S4).

### 3.5 Malnutrition Related AAMR—Stratified by Region

**FIGURE 4:**
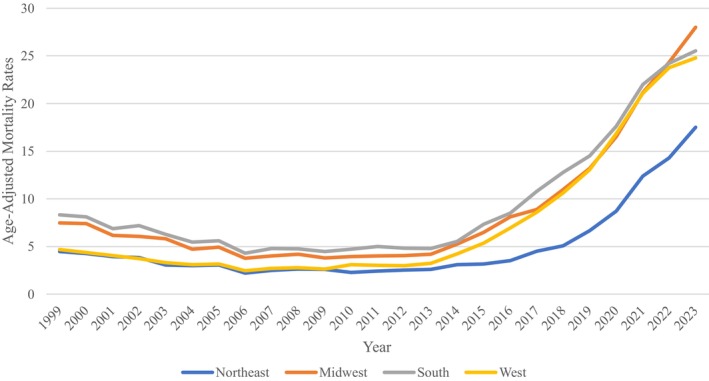
Malnutrition‐related age‐adjusted mortality rate per 100,000, stratified by census region in the United States, 1999–2023.

In the Northeast, 5 different joinpoint trends were observed. Rates declined from 1999 to 2013, yielding 2 different declining trends that were followed by an ever‐increasing trend from 2013 to 2023, with a significant spike from 2018 to 2021 with an APC of 34.3573 (95% CI: 16.7877–38.603) yielding an overall AAPC of 5.6717 (95% CI: 5.2642–6.1856) from 1999 to 2023. In the Midwest, there were 3 different joinpoints, where the trend decreased from 1999 to 2007, followed by an increasing trend till 2023, yielding an overall AAPC of 5.6867 (95% CI: 5.2479–6.2455) from 1999 to 2023. In the South region, there were 4 trends: from 1999 to 2006, the burden decreased but was then followed by an increasing trend from 2006 to 2023, with a significant trend from 2013 to 2021 with an APC of 20.6882 (95% CI: 19.6374–24.5342) that yielded an overall AAPC of 4.6558. Finally, in the West, there were 4 trends for malnutrition, with the first one decreasing, followed by three increasing trends with one that was prominent from 2013 to 2023 with an APC of 26.2260 (95% CI: 25.4662–27.1873) that resulted in the entire AAPC for the region of 7.1752 (95% CI: 6.8871–7.5162) from 1999 to 2023 (Figure 4, Figure S6, Table S5).

### 3.6 Malnutrition‐Related AAMR—Stratified by Divisions

New England observed 3 trends, where the APC declined from 1999 to 2007, followed by an increasing trend from 2007 to 2017, and then from 2017 to 2023. This yielded an overall AAPC of 6.4189 (95% CI: 5.8634–7.1134) from 1999 to 2023. Middle Atlantic also had 3 trends, an initial decreasing one from 1999 to 2010 that was followed by two increasing trends from 2010 to 2016 and then from 2016 to 2023 that resulted in an AAPC of 5.8677 (5.3314–6.5027) from 1999 to 2023 for the Middle Atlantic. However, East North Central observed four different trends from 1999 to 2023, when first a drop was observed from 1999 to 2007 that was followed by 3 increasing trends with a significant trend from 2013 to 2021 yielding an APC of 21.4295 (95% CI: 20.2573–25.0278) that resulted in an overall AAPC of 5.2962 (95% CI: 4.9396–5.733) from 1999 to 2023. West North Central observed 3 different trends, when first it declined from 1999 to 2006, which later surged to its highest values till 2023, making a final AAPC of 5.9192 (95% CI: 5.3182–6.7377) from 1999 to 2023. For South Atlantic, the trend initially declined from 1999 to 2007, which was followed by three different increasing trends from 2007 to 2023 that resulted in an overall AAPC for South Atlantic of 4.6217 (95% CI: 4.0682–5.2877) from 1999 to 2023. East South Central also had different trends with a similar first one of decreasing APC value of −6.3634 (95% CI: −13.927 to −3.6734) from 1999 to 2006 that was followed by an ever‐increasing trend from 2006 to 2023 that yielded a total AAPC of 5.4229 (95% CI: 4.938–6.0472) from 1999 to 2023. West South Central had the first declining trend from 1999 to 2006 that was followed by only two trends of increasing burden from 2006 to 2014 and then from 2014 to 2023 that resulted in an overall AAPC of 4.7322 (95% CI: 4.2706–5.3478) from 1999 to 2023. Mountains observed the highest number of trends, when the trend decreased from 1999 to 2007, that was then followed by 4 increasing trends from 2007 to 2013, 2013 to 2017, 2017 to 2021, and 2021 to 2023, that led to the final AAPC of 7.0109 (95% CI: 6.6038–7.645) from 1999 to 2023. Pacific had an initial negative APC value of −5.1807 (95% CI: −13.6263 to −1.8649) from 1999 to 2006 that then increased till 2023, yielding an overall AAPC of 7.1805 (95% CI: 6.6945–7.8991) from 1999 to 2023 (Figure S7, Table S6).

## 4 Discussion

This report shows a thorough trend analysis of age‐adjusted malnutrition‐related mortality rates in adults aged 55 and above in the United States from 1999 through 2023. The data show a significant and alarming revival of malnutrition‐related mortality among different demographic segments, although initially there were some improvements in previous years. Overall, following a trend of decline between 1999 and 2006, mortality from malnutrition started to increase substantially from 2013, with an average annual percent change (AAPC) of 5.50% (95% CI: 5.20–5.89) during the study. These trends were similar across age, gender, racial groups, and geographic areas, with remarkable variation in the magnitude and timing of increases. These shifts may represent larger systemic and policy‐level changes over time. The increase in mortality after 2013 could be related to socioeconomic stressors, healthcare access, or possible weakening of safety net policies. For example, cuts to funding for SNAP (Supplemental Nutrition Assistance Program) and senior nutrition services after austerity measures may have increased nutritional vulnerabilities for older adults (Nestle 2019). Moreover, the COVID‐19 pandemic starting in 2020 likely exacerbated food insecurity and interrupted healthcare access, leading to under‐diagnosis or inadequate management of malnutrition, particularly in long‐term care facilities and home‐bound populations. These factors may have ultimately led to the observed sharp increase in malnutrition deaths starting in 2013 (Zhu et al. 2022).

The recent resurgence of malnutrition as a mortality cause in older persons is consistent with trends in recent national data, such as a CDC‐based report from 1999 to 2020, which also noted a significant increase in mortality due to malnutrition among older persons (Mostafa et al. 2023). These results confirm our findings and highlight that malnutrition is re‐emerging as a leading cause of death in the aging U.S. population, even with the nation's high‐income status and medical advancements, where increased hospital expenditure has been linked to reduced inpatient mortality for severe medical conditions (Romley et al. 2013).

The analysis observed a consistent trend reversal in every age group (55–64, 65–74, 75–84, and ≥ 85 years) concerning mortality, with initial declines in mortality rates reversing to steep rises post‐2013. The highest change was observed in the oldest age group (≥ 85 years) with an average annual rise of 5.74%. This could be seen as the cumulative vulnerability of the elderly, where physiological, medical, and social factors interact and compound the effects of malnutrition. Malnutrition among older adults is usually more than a poor diet, but it is directly related to the aging process itself (Norman et al. 2021; Bardon et al. 2021). Additionally, malnutrition among this group is often compounded by concurrent chronic illnesses, such as dementia, cardiovascular disease, and cancer, wherein the nutritional requirement is more often elevated but difficult to satisfy. Malnutrition has been shown to weaken immune function, slow recovery from illness, prolong hospitalization stays, and precipitate a spiral of frailty and dependence (Orlandoni et al. 2017). Social determinants of health, such as social isolation, being widowed, low socioeconomic status, and inadequate support care, thus contribute to increased risk (Tomasiewicz et al. 2024).

Gender‐specific trends also yielded alarming patterns. Although females at first suffered a more severe reduction in malnutrition‐related deaths, the trend inverted after 2007, and a sharp spike was noted from 2013 to 2021. Males had a similar trend with a steep rise during the same years. These concordant rises are indicative of systemic issues within the healthcare system's ability to screen and treat malnutrition, especially in community‐living older adults who do not receive routine nutritional assessments. Women, who on average live longer than men, might be disproportionately impacted because they are more likely to live alone, have physical impairments, or experience age‐related diseases (Ostan et al. 2016; O'Connell et al. 2021; Moses et al. 2023). Another previous study demonstrated a highly significant decrease in health‐related quality of life (HRQoL) with a rising risk of malnutrition, with the effect being more notable in men than women (Kvamme et al. 2011). The gender difference may be indicative of differences in coping strategies, social support, and healthcare utilization, which could expose older men to malnutrition. These results concur with our trends as observed, in which mortality due to malnutrition increased in both genders but with different patterns, emphasizing the need for gender‐sensitive nutritional screening and intervention.

Our findings indicated that all racial and ethnic groups had initial decreases in age‐adjusted mortality rates (AAMRs) starting in 1999, but later years had significant increases, with varying intensities across groups. White people had the steepest AAPC (5.91%) in malnutrition‐attributable mortality between 1999 and 2023, reflecting a disproportionately high rate of increase in death rates. This seems to be in contrast with literature that has been reporting that Black and Hispanic populations in the U.S. consistently experience higher food insecurity rates (Gulati et al. 2025; Brown et al. 2023). As per the USDA Economic Research Service (2024), the rate of food insecurity was far greater among Black‐headed (19.8%) and Hispanic‐headed (16.2%) households than among White (8.1%) and Asian (5.4%) households. The disparity is amply documented by several national surveys and peer‐reviewed research, which explain greater food insecurity among Black people as the result of systemic disparities, residential segregation, income inequality, and restricted access to healthy food choices (Economic Research Service, n.d.). The intersection of age, race, and geography demonstrated compounding disparities. Under the mortality trend analysis, all age groups experienced rising mortality trends, but the ≥ 85 years group had the largest climbing trajectory, and the strongest trend was from White individuals. In contrast, food insecurity is often described as more prevalent among Black and Hispanic populations, suggesting that mortality risk may potentially be affected by other factors contributing to health disparities, in addition to food insecurity—chronic disease burden, healthcare utilization, and living arrangements may also afflict older White individuals with rises in mortality (Varela et al. 2023). Regionally, the Pacific and Mountain divisions experienced the greatest overall AAPCs and had a growing elderly population, with large land mass rural areas, which may further restrict nutrition and healthcare. Comprehensively evaluating these multi‐level compound disparities, indicates the need for public health interventions that are—population‐ and region specific.

Geographically, elevated trends of mortality were reported throughout all regions of the United States and all census divisions, the highest being the Pacific and Mountain divisions. Though they are economically diverse, both have registered higher housing insecurity, cost‐of‐living crises, and elderly isolation, all factors pointing toward nutritional susceptibility. Geographical disparities also highlight the effects of healthcare facilities, differing policy interventions, and socioeconomic inequality, previously highlighted in geographic health disparity studies. The Western Pacific Region has been found to experience a severe shortage of trained health workers, especially in rural and underserved communities, constraining the ability to address age‐related health conditions, such as malnutrition and frailty. Moreover, nations and regions in the Western Pacific are experiencing rapid population aging and an increasing burden of non‐communicable diseases, which can enhance the nutritional needs of older persons while at the same time overburdening healthcare systems (The Lancet Regional Health‐Western Pacific 2023, 2022). Although these studies generalize to the larger Western Pacific Region, the same patterns of aging populations, barriers to healthcare access, and chronic disease burden are seen in the U.S.

These findings necessitate increased national and regional public health policy efforts to acknowledge malnutrition as a serious, quantifiable health problem. Structured interventions such as Comprehensive Geriatric Assessment (CGA) have been demonstrated to be beneficial in enhancing outcomes for older people and can potentially reduce risks from malnutrition. A recent meta‐analysis of 29 trials involving more than 13,000 participants concluded that CGA made it more likely that older people would stay in their own homes and decreased the risk of nursing home admission, without having a significant impact on all‐cause mortality (Ellis et al. 2017). These results validate the promise of combined, multidisciplinary care in responding to functional decline and malnutrition outcomes, particularly in the oldest and most vulnerable individuals.

## 5 Limitations

This research is subject to some limitations. It is based on death certificate information, which might undercount or irregularly report malnutrition as a cause of death, especially among older people. The fact that it does not have detailed individual‐level information, including socioeconomic status, comorbidities, or care setting, precludes adjustment for important risk factors. Regional trends can also hide important differences. Moreover, temporal fluctuations in coding strategies and clinical acknowledgement may have also affected the witnessed trends. Finally, the investigation fails to differentiate between causes or types of malnutrition; therefore, it is problematic to determine drivers of higher mortality.

## 6 Conclusion

This study provides a comprehensive analysis of malnutrition‐related mortality trends in older adults in the United States from 1999 to 2023. The alarming resurgence in malnutrition‐related deaths, particularly after 2013, underscores the critical need for targeted public health interventions. Significant disparities were noted, with higher rises in mortality among the Whites, males, and the age group of 65–74 and 85+ years. To address these challenges, structured interventions such as Comprehensive Geriatric Assessment (CGA) have shown promise in reducing malnutrition risks and improving health outcomes. By implementing gender‐sensitive and culturally appropriate nutritional screening and management strategies, healthcare systems can better meet the needs of this vulnerable population.

Furthermore, addressing the social determinants of health, such as social isolation, economic insecurity, and inadequate access to healthcare, is crucial in mitigating malnutrition among older adults. Enhanced public health policies and regional efforts are necessary to recognize and manage malnutrition as a serious health problem, ensuring that older adults receive the support and care they need to lead healthy and dignified lives.

The findings from this study serve as a call to action for healthcare providers, policymakers, and community leaders to prioritize malnutrition prevention and intervention for older adults, ultimately aiming to improve their quality of life and reduce the mortality burden associated with malnutrition.

## Author Contributions

**Shree Rath:** conceptualization (lead), data curation (lead), investigation (lead), methodology (lead), project administration (lead), resources (lead), supervision (lead), validation (lead), writing – original draft (equal), writing – review and editing (lead). **Ghazal Ishaque:** writing – original draft (lead). **Saif Ur Rahman:** data curation (equal), formal analysis (lead), resources (lead), software (lead). **Ahmad Sanan:** writing – original draft (equal). **Bushra Syed:** writing – original draft (equal), writing – review and editing (equal). **Mubashira Ali:** writing – original draft (equal). **Pinkey Kumari:** writing – original draft (equal). **Amar Lal:** project administration (equal), resources (equal), supervision (equal), writing – review and editing (equal).

## Ethics Statement

The authors have nothing to report.

## Consent

The authors have nothing to report. All authors have reviewed the final draft and agree to the publication of the manuscript in its current form.

## Conflicts of Interest

The authors declare no conflicts of interest.

## Supporting information


**Figure S1:** Overall Malnutrition‐Related Annual Percent Change (APC) per 100,000 in the United States, 1999–2023.
**Figure S2:** Malnutrition‐related Annual Percent Change (APC) per 100,000, Stratified by Ten‐year Age Groups in the United States, 1999–2023.
**Figure S3:** Malnutrition‐related Annual Percent Change (APC) per 100,000, Stratified by Gender in the United States, 1999–2023.
**Figure S4:** Malnutrition‐related Annual Percent Change (APC) per 100,000, Stratified by Race in the United States, 1999–2023.
**Figure S6:** Malnutrition‐related Annual Percent Change (APC) per 100,000, Stratified by Census Regions in the United States, 1999–2023.
**Figure S7:** Malnutrition‐related Annual Percent Change (APC) per 100,000, Stratified by Census Division in the United States, 1999–2023.
**Table S1:** Overall, Sex‐stratified and Race‐stratified Malnutrition‐related deaths per 100,000 in the United States from 1999 to 2023.
**Table S2:** Overall and Sex‐stratified Malnutrition‐related AAMR per 100,000 in the United States from 1999 to 2023.
**Table S3:** Malnutrition‐related deaths per 100,000 stratified by ten‐year age groups in the United States from 1999 to 2023
**Table S4:** Race‐stratified Malnutrition‐related AAMR per 100,000 in the United States from 1999 to 2023
**Table S5:** Census Region stratified Malnutrition‐related AAMR per 100,000 in the United States from 1999 to 2023.
**Table S6:** Census Division stratified Malnutrition‐related AAMR per 100,000 in the United States from 1999 to 2023.

## Data Availability

The data that supports the findings of this study is available in the Supporting Information of this article.
