# Supplementary material for: Rising Deaths due to Malnutrition and Growing Disparities in the U.S.: A 24‐Year Trend Analysis From 1999 and 2023
Source: Food Sci Nutr. 2025 Oct 22;13(10):e71040. doi: 10.1002/fsn3.71040 (PMC12542810; doi:10.1002/fsn3.71040)
Supplement: Supplementary file 1 — Figure S1: Overall Malnutrition‐Related Annual Percent Change (APC) per 100,000 in the United States, 1999–2023. Figure S2: Malnutrition‐related Annual Percent Change (APC) per 100,000, Stratified by Ten‐year Age Groups in the United States, 1999–2023. Figure S3: Malnutrition‐related Annual Percent Change (APC) per 100,000, Stratified by Gender in the United States, 1999–2023. Figure S4: Malnutrition‐related Annual Percent Change (APC) per 100,000, Stratified by Race in the United States, 1999–2023. Figure S6: Malnutrition‐related Annual Percent Change (APC) per 100,000, Stratified by Census Regions in the United States, 1999–2023. Figure S7: Malnutrition‐related Annual Percent Change (APC) per 100,000, Stratified by Census Division in the United States, 1999–2023. Table S1: Overall, Sex‐stratified and Race‐stratified Malnutrition‐related deaths per 100,000 in the United States from 1999 to 2023. Table S2: Overall and Sex‐stratified Malnutrition‐related AAMR per 100,000 in the United States from 1999 to 2023. Table S3: Malnutrition‐related deaths per 100,000 stratified by ten‐year age groups in the United States from 1999 to 2023 Table S4: Race‐stratified Malnutrition‐related AAMR per 100,000 in the United States from 1999 to 2023 Table S5: Census Region stratified Malnutrition‐related AAMR per 100,000 in the United States from 1999 to 2023. Table S6: Census Division stratified Malnutrition‐related AAMR per 100,000 in the United States from 1999 to 2023. [file FSN3-13-e71040-s001.docx]

**Supplementary Material**

| Year | Overall | Male | Female | NH American Indian or Alaska Native | NH Black or African American | NH White | Hispanic or Latino | Population |
| --- | --- | --- | --- | --- | --- | --- | --- | --- |
| 1999 | 3797 | 1281 | 2516 | 25 | 499 | 3230 | 122 | 58575867 |
| 2000 | 3744 | 1226 | 2518 | 24 | 455 | 3226 | 126 | 59266437 |
| 2001 | 3290 | 1068 | 2222 | 26 | 391 | 2829 | 122 | 60395586 |
| 2002 | 3341 | 1152 | 2189 | 21 | 434 | 2852 | 125 | 62225539 |
| 2003 | 3005 | 1017 | 1988 | 18 | 401 | 2554 | 106 | 63872474 |
| 2004 | 2677 | 945 | 1732 | 22 | 353 | 2258 | 96 | 65508623 |
| 2005 | 2823 | 981 | 1842 | 18 | 365 | 2389 | 122 | 67291295 |
| 2006 | 2213 | 771 | 1442 | 13 | 278 | 1884 | 105 | 69094220 |
| 2007 | 2489 | 916 | 1573 | 22 | 340 | 2077 | 96 | 70954145 |
| 2008 | 2576 | 971 | 1605 | 19 | 308 | 2205 | 132 | 72934684 |
| 2009 | 2494 | 946 | 1548 | 19 | 304 | 2121 | 113 | 75028775 |
| 2010 | 2627 | 1009 | 1618 | 27 | 318 | 2236 | 148 | 76750713 |
| 2011 | 2834 | 1098 | 1736 | 35 | 341 | 2404 | 145 | 79456281 |
| 2012 | 2857 | 1085 | 1772 | 22 | 346 | 2432 | 150 | 81731558 |
| 2013 | 3012 | 1181 | 1831 | 24 | 384 | 2536 | 155 | 84020505 |
| 2014 | 3743 | 1464 | 2279 | 31 | 457 | 3158 | 184 | 86320792 |
| 2015 | 4797 | 1773 | 3024 | 36 | 572 | 4080 | 270 | 88638671 |
| 2016 | 5915 | 2235 | 3680 | 43 | 675 | 5061 | 326 | 90707339 |
| 2017 | 7379 | 2676 | 4703 | 44 | 860 | 6284 | 429 | 92854337 |
| 2018 | 9038 | 3343 | 5695 | 52 | 1053 | 7726 | 534 | 94703829 |
| 2019 | 10949 | 4067 | 6882 | 63 | 1250 | 9390 | 650 | 96506800 |
| 2020 | 13960 | 5150 | 8810 | 98 | 1541 | 11977 | 840 | 98063042 |
| 2021 | 16657 | 6214 | 10443 | 89 | 1733 | 14396 | 973 | 98651017 |
| 2022 | 20110 | 7354 | 12756 | 109 | 1918 | 17550 | 1116 | 99880289 |
| 2023 | 21790 | 8048 | 13742 | 112 | 2093 | 18973 | 1231 | 101102772 |

**Supplementary table 1.** Overall, Sex-stratified and Race-stratified Malnutrition-related deaths per 100,000 in the United States from 1999 to 2023

| Age-Adjusted Rate (95% CI) | | | |
| --- | --- | --- | --- |
| Year | **Male** | **Female** | **Overall** |
| 1999 | 6.30 | 6.61 | 6.55 |
| 2000 | 5.88 | 6.56 | 6.39 |
| 2001 | 5.02 | 5.69 | 5.52 |
| 2002 | 5.36 | 5.58 | 5.53 |
| 2003 | 4.55 | 4.99 | 4.88 |
| 2004 | 4.11 | 4.35 | 4.27 |
| 2005 | 4.17 | 4.54 | 4.43 |
| 2006 | 3.12 | 3.47 | 3.36 |
| 2007 | 3.63 | 3.74 | 3.73 |
| 2008 | 3.66 | 3.76 | 3.76 |
| 2009 | 3.51 | 3.59 | 3.55 |
| 2010 | 3.62 | 3.70 | 3.67 |
| 2011 | 3.80 | 3.89 | 3.85 |
| 2012 | 3.62 | 3.86 | 3.77 |
| 2013 | 3.81 | 3.86 | 3.89 |
| 2014 | 4.61 | 4.72 | 4.71 |
| 2015 | 5.46 | 6.12 | 5.84 |
| 2016 | 6.80 | 7.32 | 7.10 |
| 2017 | 7.89 | 9.10 | 8.66 |
| 2018 | 9.66 | 10.87 | 10.40 |
| 2019 | 11.40 | 12.94 | 12.39 |
| 2020 | 14.14 | 16.33 | 15.53 |
| 2021 | 17.98 | 20.75 | 19.74 |
| 2022 | 20.20 | 23.45 | 22.23 |
| 2023 | 21.74 | 25.96 | 24.37 |

**Supplementary Table 2.** Overall and Sex-stratified Malnutrition-related AAMR per 100,000 in the United States from 1999 to 2023

| Year | Ten-Year Age Groups | | | |
| --- | --- | --- | --- | --- |
|  | **55-64 years** | **65-74 years** | **75-84 years** | **85+ years** |
| 1999 | 154 | 407 | 1159 | 2077 |
| 2000 | 158 | 423 | 1199 | 1964 |
| 2001 | 146 | 347 | 1025 | 1772 |
| 2002 | 156 | 354 | 1067 | 1764 |
| 2003 | 160 | 320 | 952 | 1573 |
| 2004 | 152 | 288 | 864 | 1373 |
| 2005 | 149 | 333 | 866 | 1475 |
| 2006 | 173 | 262 | 673 | 1105 |
| 2007 | 188 | 314 | 755 | 1232 |
| 2008 | 197 | 350 | 760 | 1269 |
| 2009 | 224 | 314 | 743 | 1213 |
| 2010 | 231 | 366 | 772 | 1258 |
| 2011 | 262 | 413 | 827 | 1332 |
| 2012 | 246 | 409 | 823 | 1379 |
| 2013 | 266 | 458 | 833 | 1455 |
| 2014 | 349 | 548 | 974 | 1872 |
| 2015 | 339 | 670 | 1176 | 2612 |
| 2016 | 421 | 747 | 1437 | 3310 |
| 2017 | 471 | 998 | 1735 | 4175 |
| 2018 | 492 | 1141 | 2239 | 5166 |
| 2019 | 631 | 1326 | 2725 | 6267 |
| 2020 | 751 | 1818 | 3475 | 7916 |
| 2021 | 821 | 2112 | 4238 | 9486 |
| 2022 | 945 | 2632 | 5366 | 11167 |
| 2023 | 974 | 2834 | 5828 | 12154 |

**Supplementary Table 3.** Malnutrition-related deaths per 100,000 stratified by ten-year age groups in the United States from 1999 to 2023

|  | Age-Adjusted Rate (95% CI) | | | |
| --- | --- | --- | --- | --- |
| Year | **NH American Indian or Alaska Native** | **NH Black or African American** | **NH White** | **Hispanic or Latino** |
| 1999 | 11.29 | 10.95 | 6.19 | 5.42 |
| 2000 | 10.79 | 9.82 | 6.13 | 5.19 |
| 2001 | 10.38 | 8.45 | 5.28 | 4.95 |
| 2002 | 9.25 | 9.25 | 5.26 | 4.65 |
| 2003 | - | 8.49 | 4.61 | 3.74 |
| 2004 | 8.27 | 7.31 | 4.04 | 3.16 |
| 2005 | - | 7.28 | 4.17 | 3.86 |
| 2006 | - | 5.37 | 3.22 | 3.14 |
| 2007 | 6.73 | 6.43 | 3.48 | 2.66 |
| 2008 | - | 5.61 | 3.66 | 3.36 |
| 2009 | - | 5.38 | 3.42 | 2.76 |
| 2010 | 7.10 | 5.39 | 3.58 | 3.42 |
| 2011 | 8.23 | 5.58 | 3.72 | 3.08 |
| 2012 | 4.77 | 5.51 | 3.67 | 2.98 |
| 2013 | 5.18 | 5.70 | 3.76 | 2.93 |
| 2014 | 7.12 | 6.59 | 4.55 | 3.27 |
| 2015 | 7.08 | 8.10 | 5.79 | 4.57 |
| 2016 | 7.72 | 9.30 | 7.03 | 5.31 |
| 2017 | 7.43 | 11.33 | 8.55 | 6.43 |
| 2018 | 9.14 | 13.67 | 10.35 | 7.66 |
| 2019 | 9.97 | 15.61 | 12.38 | 9.03 |
| 2020 | 14.44 | 18.64 | 15.64 | 11.05 |
| 2021 | 14.10 | 22.15 | 20.28 | 13.31 |
| 2022 | 16.05 | 23.43 | 23.08 | 14.36 |
| 2023 | 15.79 | 25.22 | 25.35 | 15.48 |

**Supplementary Table 4.** Race-stratified Malnutrition-related AAMR per 100,000 in the United States from 1999 to 2023

| Age-Adjusted Rate (95% CI) | | | | |
| --- | --- | --- | --- | --- |
| Year | **Census Region 1: Northeast** | **Census Region 2: Midwest** | **Census Region 3: South** | **Census Region 4: West** |
| 1999 | 4.46 | 7.48 | 8.31 | 4.67 |
| 2000 | 4.25 | 7.41 | 8.12 | 4.36 |
| 2001 | 3.94 | 6.18 | 6.88 | 4.04 |
| 2002 | 3.83 | 6.08 | 7.19 | 3.74 |
| 2003 | 3.06 | 5.83 | 6.29 | 3.31 |
| 2004 | 2.99 | 4.72 | 5.45 | 3.09 |
| 2005 | 3.05 | 4.94 | 5.59 | 3.15 |
| 2006 | 2.23 | 3.76 | 4.31 | 2.44 |
| 2007 | 2.48 | 4.02 | 4.78 | 2.70 |
| 2008 | 2.62 | 4.18 | 4.74 | 2.77 |
| 2009 | 2.59 | 3.82 | 4.47 | 2.65 |
| 2010 | 2.28 | 3.95 | 4.74 | 3.09 |
| 2011 | 2.42 | 4.03 | 5.01 | 3.04 |
| 2012 | 2.53 | 4.05 | 4.82 | 2.98 |
| 2013 | 2.59 | 4.18 | 4.79 | 3.24 |
| 2014 | 3.09 | 5.25 | 5.54 | 4.22 |
| 2015 | 3.16 | 6.47 | 7.32 | 5.36 |
| 2016 | 3.53 | 8.10 | 8.51 | 6.94 |
| 2017 | 4.52 | 8.90 | 10.79 | 8.62 |
| 2018 | 5.09 | 10.98 | 12.79 | 10.61 |
| 2019 | 6.68 | 13.19 | 14.51 | 13.05 |
| 2020 | 8.71 | 16.52 | 17.63 | 16.85 |
| 2021 | 12.37 | 21.16 | 21.99 | 21.07 |
| 2022 | 14.30 | 24.34 | 24.21 | 23.78 |
| 2023 | 17.51 | 28.00 | 25.52 | 24.80 |

**Supplementary Table 5.** Census Region stratified Malnutrition-related AAMR per 100,000 in the United States from 1999 to 2023

| Year | Age Adjusted Mortality Rate | | | | | | | | |
| --- | --- | --- | --- | --- | --- | --- | --- | --- | --- |
|  | **New England** | **Middle Atlantic** | **East North Central** | **West North Central** | **South Atlantic** | **East South Central** | **West South Central** | **Mountain** | **Pacific** |
| 1999 | 3.96 | 4.69 | 7.76 | 7.00 | 6.93 | 8.58 | 10.88 | 7.53 | 3.53 |
| 2000 | 4.15 | 4.27 | 7.35 | 7.43 | 6.74 | 9.36 | 10.05 | 7.72 | 3.08 |
| 2001 | 3.22 | 4.21 | 6.32 | 5.79 | 5.69 | 8.14 | 8.50 | 6.90 | 2.93 |
| 2002 | 3.78 | 3.90 | 6.40 | 5.40 | 6.00 | 8.54 | 8.85 | 5.46 | 3.10 |
| 2003 | 3.16 | 3.03 | 6.10 | 5.19 | 5.55 | 6.98 | 7.46 | 4.85 | 2.63 |
| 2004 | 2.79 | 3.10 | 5.10 | 3.84 | 4.63 | 6.42 | 6.65 | 4.37 | 2.62 |
| 2005 | 2.91 | 3.10 | 5.40 | 4.04 | 4.24 | 6.85 | 7.72 | 4.61 | 2.58 |
| 2006 | 2.54 | 2.15 | 3.96 | 3.16 | 3.23 | 5.22 | 6.05 | 3.61 | 2.03 |
| 2007 | 2.21 | 2.63 | 4.20 | 3.62 | 3.49 | 6.10 | 6.80 | 3.19 | 2.53 |
| 2008 | 2.54 | 2.64 | 4.55 | 3.37 | 3.34 | 5.94 | 6.94 | 3.15 | 2.59 |
| 2009 | 3.01 | 2.48 | 4.03 | 3.31 | 3.29 | 5.71 | 6.35 | 3.64 | 2.29 |
| 2010 | 2.15 | 2.28 | 4.30 | 3.17 | 3.53 | 5.34 | 6.91 | 4.03 | 2.65 |
| 2011 | 2.74 | 2.30 | 4.21 | 3.65 | 3.58 | 5.73 | 7.52 | 3.95 | 2.70 |
| 2012 | 2.94 | 2.34 | 4.15 | 3.85 | 3.27 | 6.07 | 7.31 | 3.77 | 2.65 |
| 2013 | 2.72 | 2.58 | 4.37 | 3.73 | 3.39 | 5.51 | 7.24 | 4.02 | 2.92 |
| 2014 | 3.24 | 3.00 | 5.51 | 4.63 | 4.61 | 5.46 | 7.53 | 5.36 | 3.73 |
| 2015 | 3.48 | 3.08 | 6.90 | 5.57 | 6.19 | 7.47 | 9.61 | 7.95 | 4.26 |
| 2016 | 3.89 | 3.48 | 8.54 | 7.09 | 7.73 | 8.81 | 9.97 | 10.32 | 5.48 |
| 2017 | 3.87 | 4.70 | 9.40 | 7.81 | 9.72 | 11.62 | 12.54 | 13.98 | 6.29 |
| 2018 | 4.45 | 5.33 | 11.69 | 9.49 | 11.16 | 15.96 | 14.50 | 17.99 | 7.45 |
| 2019 | 6.11 | 6.91 | 14.30 | 10.83 | 12.68 | 17.62 | 16.68 | 24.17 | 8.10 |
| 2020 | 8.42 | 8.79 | 17.09 | 15.34 | 15.54 | 21.69 | 19.80 | 30.67 | 10.59 |
| 2021 | 11.57 | 12.66 | 21.31 | 20.89 | 19.72 | 25.68 | 24.86 | 38.81 | 13.04 |
| 2022 | 14.01 | 14.40 | 23.97 | 25.19 | 21.37 | 28.64 | 27.91 | 41.23 | 15.82 |
| 2023 | 18.56 | 17.14 | 27.12 | 29.97 | 21.29 | 32.78 | 30.78 | 40.49 | 17.44 |

**Supplementary Table 6.** Census Division stratified Malnutrition-related AAMR per 100,000 in the United States from 1999 to 2023


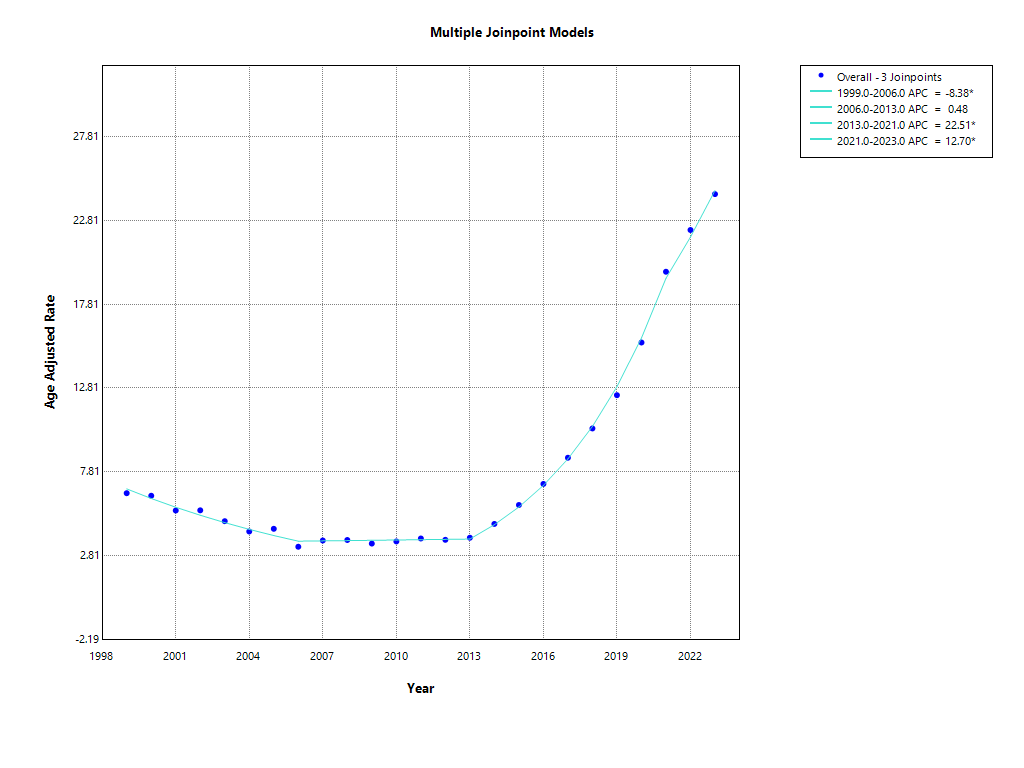


Supplemental Figure 1: Overall Malnutrition-Related Annual Percent Change (APC) per 100,000 in the United States, 1999-2023


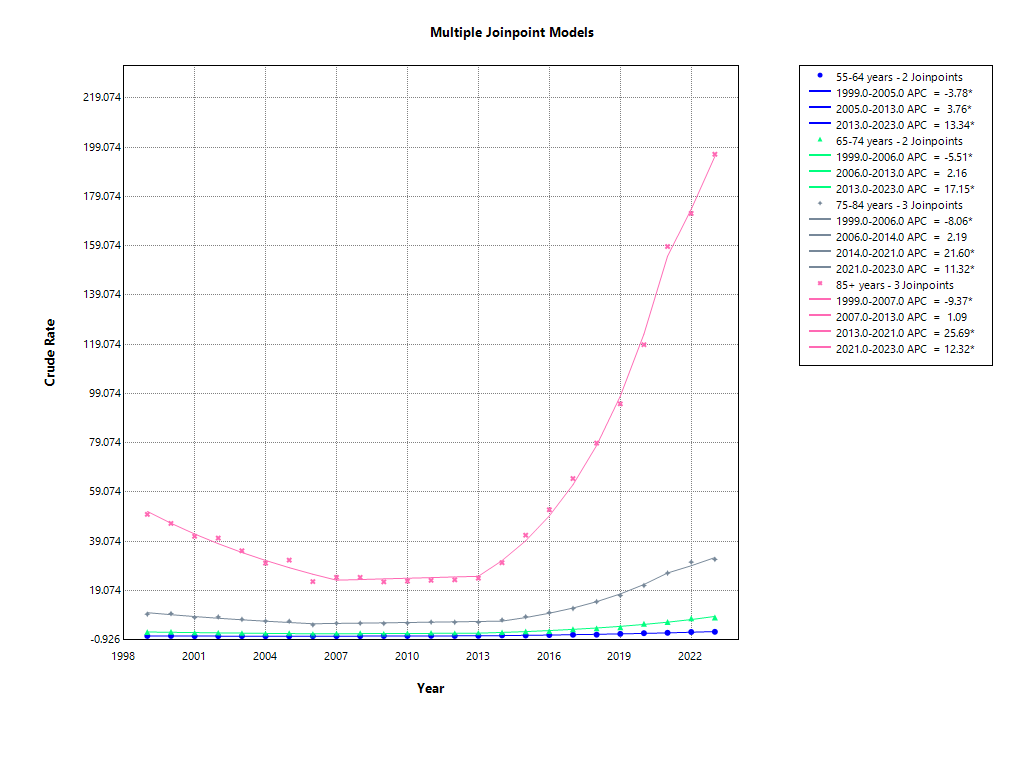


Supplemental Figure 2: Malnutrition-related Annual Percent Change (APC) per 100,000, Stratified by Ten-year Age Groups in the United States, 1999-2023


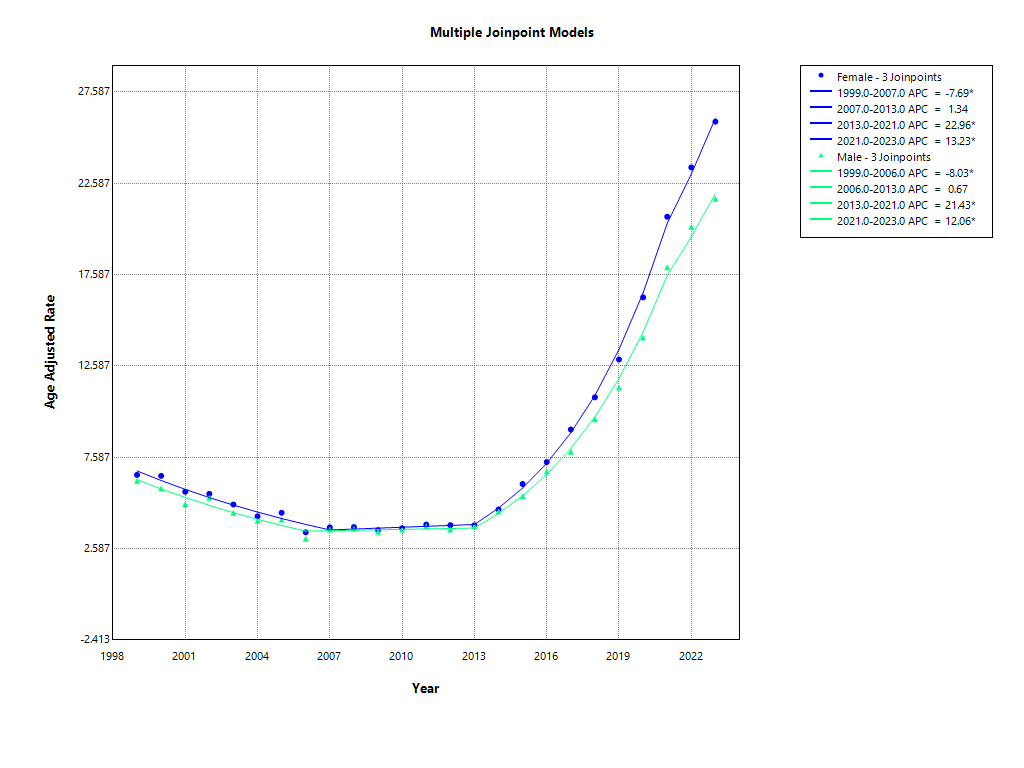


Supplemental Figure 3: Malnutrition-related Annual Percent Change (APC) per 100,000, Stratified by Gender in the United States, 1999-2023


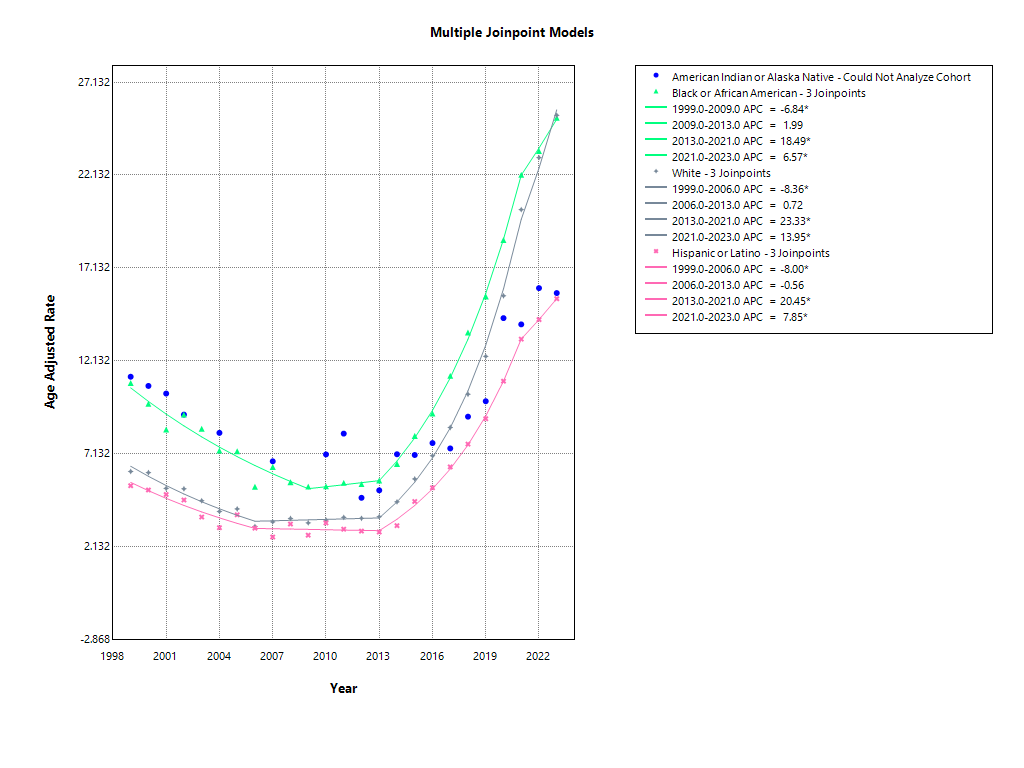


Supplemental Figure 4: Malnutrition-related Annual Percent Change (APC) per 100,000, Stratified by Race in the United States, 1999-2023


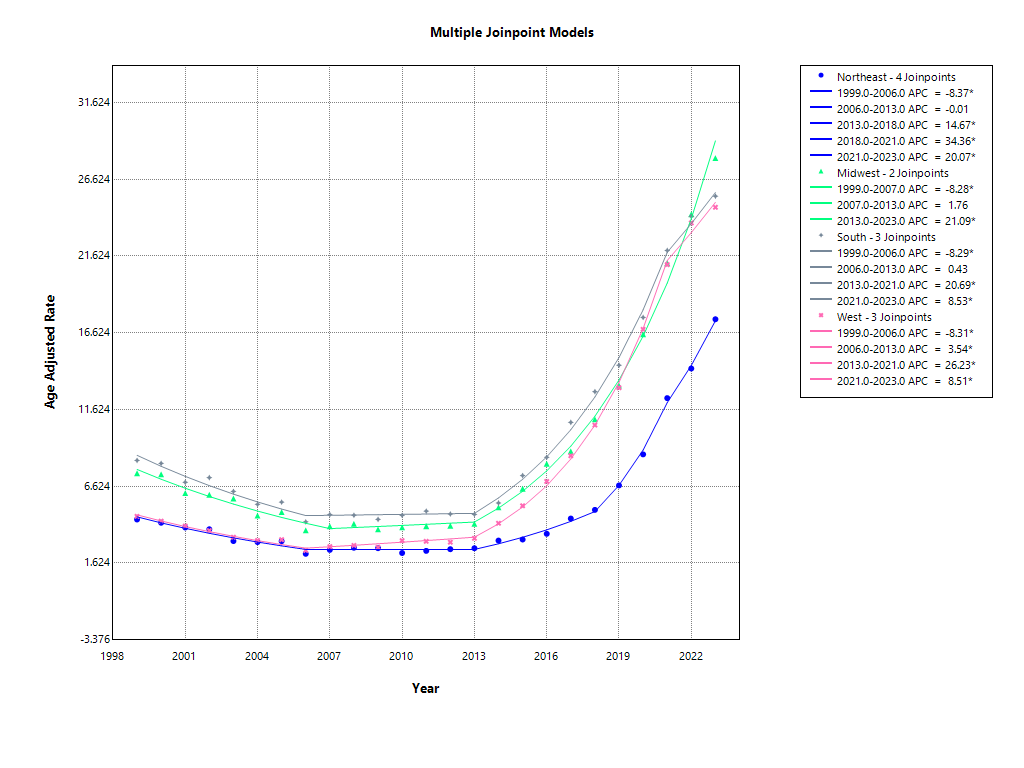


Supplemental Figure 6: Malnutrition-related Annual Percent Change (APC) per 100,000, Stratified by Census Regions in the United States, 1999-2023


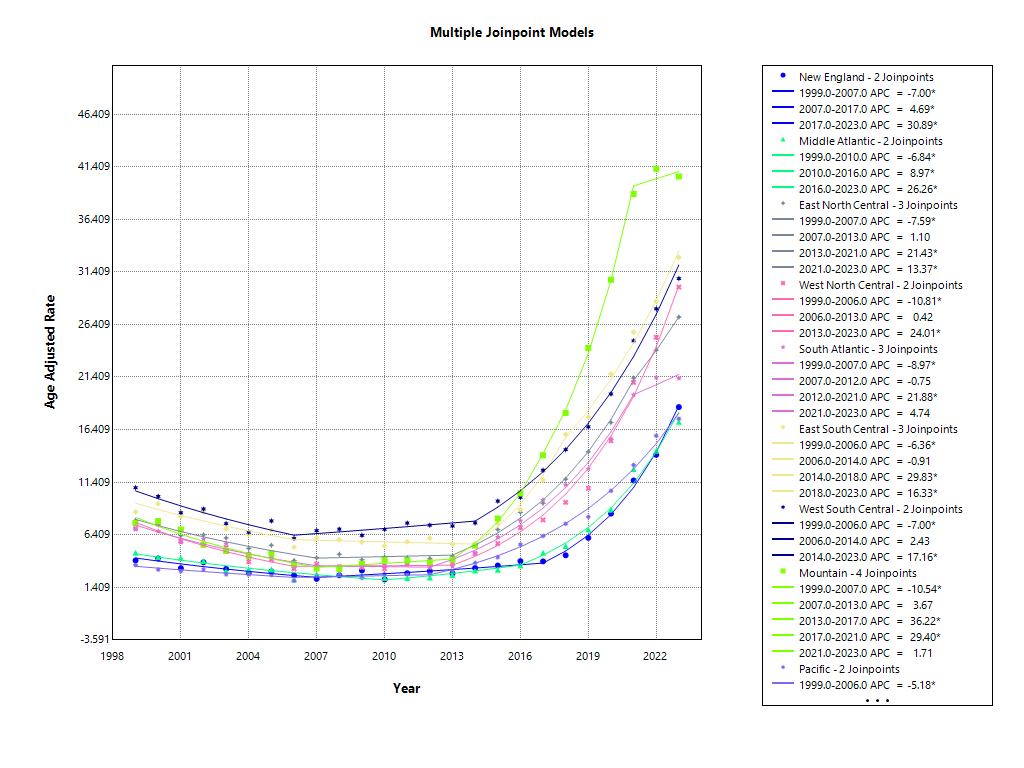


Supplemental Figure 7: Malnutrition-related Annual Percent Change (APC) per 100,000, Stratified by Census Division in the United States, 1999-2023
